# ASSOCIATIONS BETWEEN LIFE SPACE MOBILITY AND ASPECTS OF HOUSING AND SERVICE INFRASTRUCTURE

**DOI:** 10.1093/geroni/igac059.1242

**Published:** 2022-12-20

**Authors:** Bjorn Slaug, Magnus Zingmark, Susanne Iwarsson

**Affiliations:** Lund University, Lund, Skane Lan, Sweden; Lund University, Lund, Skane Lan, Sweden; Lund University, Lund, Skane Lan, Sweden

## Abstract

To achieve the widespread policy goal of active aging, it is central that older adults can participate and be independent in activities both in and outside the home. Life-space mobility refers to the extent of the area where a person performs different activities, including frequency and level of independence, and is considered an indicator of active aging. There is, however, a lack of studies on the relationship between life-space mobility and aspects of the home and neighborhood environment. The aim of this study was to explore associations between life-space mobility and (a) perceived usability of the home and (b) satisfaction with commercial and societal services in the surrounding neighborhood. We utilized the baseline survey of the Swedish RELOC-AGE project (N=1,964; mean age=69; 45% men), comprising data from adults aged 55+ with an interest for relocation. We found weak but significant correlations between life-space mobility and usability of the home (r=0.10; p< 0.001), and between life-space mobility and satisfaction with service infrastructure (r=0.09; p< 0.001). The correlations were stronger for men and for adults aged 65-74 years, compared to younger and older age groups. Those with their independent life space limited to the home and the close exterior surroundings (balcony, garden etc) assessed the usability of the home significantly lower (p=0.04) than those with more extended independent life space. There was no difference with regard to infrastructure satisfaction. These results suggest that improvement of both the home environment and service infrastructure may be important in supporting extended life space mobility among older adults.

